# Crystal Structure of Transglutaminase 2 with GTP Complex and Amino Acid Sequence Evidence of Evolution of GTP Binding Site

**DOI:** 10.1371/journal.pone.0107005

**Published:** 2014-09-05

**Authors:** Tae-Ho Jang, Dong-Sup Lee, Kihang Choi, Eui Man Jeong, In-Gyu Kim, Young Whan Kim, Jung Nyeo Chun, Ju-Hong Jeon, Hyun Ho Park

**Affiliations:** 1 School of Biotechnology, Yeungnam University, Gyeongsan, South Korea; 2 Graduate School of Biochemistry, Yeungnam University, Gyeongsan, South Korea; 3 Department of Physiology and Biophysics at Seoul National University College of Medicine, Seoul, South Korea; 4 Department of Chemistry, Korea University, Seoul, South Korea; 5 Department of Biochemistry and Molecular Biology/Aging and Apoptosis Research Center (AARC), Seoul National University College of Medicine, Seoul, South Korea; 6 Division of Pulmonary and Critical Care Medicine, Department of Internal Medicine and Lung Institute of Medical Research Center, Seoul National University College of Medicine, Seoul, South Korea; 7 Department of Physiology, Department of Biomedical Sciences, and Institute of Dermatological Science, Seoul National University College of Medicine, Seoul, South Korea; University of Texas MD Anderson Cancer Center, United States of America

## Abstract

Transglutaminase2 (TG2) is a multi-functional protein involved in various cellular processes, including apoptosis, differentiation, wound healing, and angiogenesis. The malfunction of TG2 causes many human disease including inflammatory disease, celiac disease, neurodegenerative diseases, tissue fibrosis, and cancers. Protein cross-linking activity, which is representative of TG2, is activated by calcium ions and suppressed by GTP. Here, we elucidated the structure of TG2 in complex with its endogenous inhibitor, GTP. Our structure showed why GTP is the optimal nucleotide for interacting with and inhibiting TG2. In addition, sequence comparison provided information describing the evolutionary scenario of GTP usage for controlling the activity of TG2.

## Introduction

Transglutaminase2 (TG2) is a multi-functional protein that exerts protein cross-linking activity [1]. It is well known that various TG2 activities are involved in many important cellular processes, including apoptosis [2]–[4], angiogenesis [5], [6], wound healing [6], [7], and neuronal regeneration [8], and bone development. TG2 has gained attention because it is linked to many human diseases, including inflammatory disease [9], celiac disease [10], neurodegenerative disease [11], [12], diabetes [13], tissue fibrosis [14], and cancers [15]–[17]. TG2 involvement in cancer is particularly well known owing to several studies that showed that TG2 down-regulation is detected in aggressive tumors, while transfection of recombinant TG2 applied to several tumor cells led to significant reduction of tumor progression [5], [16], [18], [19]. Additionally, alzheimer’s disease (AD)-related proteins including tau, Aβ, and α-synuclein were cross-linked and accumulated by overexpressed TG2 in the pathological stage of this disease [20]–[23]. TG2 is located in the extracellular part, cytosol, and nucleus of cells [24]. In the cytosol, TG2 functions as a signal transfer molecule that transmits a receptor signal to an effector by binding GTP and hydrolyzing it to GDP and Pi [25]. When secreted outside the cell, TG2 functions as a cross-linking enzyme in the matrix [26]. Protein cross-linking activity of TG2 is positively controlled by calcium and negatively regulated by GTP [27]. In mammals, eight different TG isoenzymes have been identified at the genomic level [28]. Although the substrate specificity and mechanism of activity regulation vary significantly within this enzyme family, all members share a high sequence identity and structural similarity.

Human TG2, the most ubiquitous isoform of the TG family, is 687 amino acids in length and consists of four domains, an N-terminal β-sandwich domain, catalytic domain and two C-terminal β-barrel domains [29]. Three crystal structures that are complexed with guanosine diphosphate (GDP), adenosine triphosphate (ATP), and irreversible inhibitor, have been elucidated to date [29]–[31]. These structures showed that TG2 undergoes a large conformational change upon binding of irreversible inhibitor at the active site. GTP inactivates TG2 by promoting transition to the compact conformation, while calcium activates TG2 via an unknown mechanism. It is believed that extracellular TG2 is maintained in a closed conformation as a result of GTP binding. However, circumstances in which Ca^2+^ is increased trigger rapid activation of TG2 into an open conformation. A unique GTP binding site is located in a cleft between the catalytic core and the first β-barrel domain.

In the current study, we elucidated the structure of TG2 in complex with its inhibitor, GTP. Our structure showed a conserved GTP binding cleft formed by Phe174, Ser482, Met483, Arg476, Arg478, Arg580, and Tyr583 on TG2. The structure of the GTP-bound form of TG2 indicates that Ser482 is critical to accommodation of GTP, not ATP, via formation of an additional H-bond with GTP. Our structure also revealed that the side chain of Arg478 can be flexible to accommodate either GTP or GDP. In addition, sequence comparison with the inter-isotype TG family and inter-species TG2 protein revealed information pertaining to evolutionary GTP usage for controlling the activity of TG2.

## Materials and Methods

### Protein expression and purification

Full-length human TG2 corresponding to amino acids 1–687 cloned in pOKD homemade vector [32] was transformed into BL21 (DE3) *E. coli* competent cells. The expression was then induced by treating the bacteria with 0.125 mM isopropyl β-D-thiogalactopyranoside (IPTG) for 25 h at 18°C. Cells expressing full-length human TG2 were pelleted by centrifugation, resuspended and lysed by sonication in 50 ml lysis buffer (50 mM sodium-phosphate buffer at pH 7.5, 400 mM NaCl, 5 mM benzamidine, 1 mM 2-mercaptoethanol, 50 µM GTP, 1 mM PMSF, 0.5% (v/v) triton X-100 and 5 mM imidazole). The lysate was then centrifuged at 16,000 rpm for 30 min at 4°C, after which the supernatant fractions were applied to a gravity-flow column (BioRad) packed with Ni-NTA affinity resin (Qiagen). Next, the unbound bacterial proteins were removed from the column using washing buffer (50 mM sodium-phosphate buffer at pH 7.5, 400 mM NaCl, 5 mM benzamidine, 1 mM 2-mercaptoethanol, 50 µM GTP, 1 mM PMSF, 0.5% (v/v) triton X-100 and 5 mM imidazole). The C-terminal His-tagged TG2 was eluted from the column using elution buffer (50 mM HEPES buffer at pH 7.0, 100 mM NaCl, 50 µM GTP, 10% (v/v) glycerol and 300 mM imidazole). The elution fractions were then collected at a 0.5 ml scale to 4 ml. Next, the collected TG2 was applied to a Superdex 200 gel filtration column (GE healthcare) that had been pre-equilibrated with a solution of 20 mM Tris at pH 8.0 and 150 mM NaCl. The purified TG2 was subsequently applied to a mono Q ion-exchange column (GE Healthcare) using starting buffer (20 mM Tris pH 8.0) and elution buffer (20 mM Tris pH 8.0, 1 M NaCl). Finally, the eluted TG2 was applied to a Superdex 200 gel filtration column (GE healthcare) that had been pre-equilibrated with a solution of 20 mM Tris at pH 8.0 and 150 mM NaCl. The purified TG2 was collected and concentrated to 10–12 mg ml^−1^. The peak was then confirmed to contain TG2 by SDS-PAGE.

### Crystallization and data collection

The crystallization conditions were initially screened at 293 K by the hanging-drop vapor-diffusion method. Crystals were grown on plates by equilibrating a mixture containing 1 µl protein solution and 1 µl of reservoir solution containing 20 mM Mes at pH 6.8, 200 mM sodium chloride, 20 mM MgCl^2^, 6% PEG 3350, 5 mM DTT, and 24% glycerol against 0.4 ml of reservoir solution, which is similar to previously employed conditions [29], [30]. A 2.8 Å native diffraction data set was collected from a single crystal at beamline BL-4A of the Pohang Accelerator Laboratory (PAL), South Korea. The data sets were indexed and processed using HKL2000.

### Structure determination and analysis

The structure was determined by the molecular replacement phasing method using *Phaser* [33]. One chain of the previously solved structure of ATP-bound TG2 (PDB code: 3LY6) [30] was used as a search model. Model building and refinement were conducted by COOT [34] and Refmac5 [35], respectively. Water molecules were added using the ARP/wARP function in Refmac5. The geometry was inspected using PROCHECK and found to be reasonable. A total of 86.63% of the amino acids were located in the most favorable region, while 13.37% were in the allowed regions of the Ramachandran plot. All molecular figures were generated using the program Pymol [36].

### MALS

The absolute molar mass of the TG2 was determined by multi angle light scattering (MALS). Briefly, the target protein was loaded onto a Superdex 200 HR 10/30 gel-filtration column (GE Healthcare) that had been pre-equilibrated in a buffer containing 20 mM Tris-HCl pH 8.0 and 150 mM NaCl. The acta chromatography system (GE Healthcare) was coupled to a MALS detector (mini-DAWM treos) and a refractive index detector (Optilab DSP) (Wyatt Technology).

### Sequence alignment

The amino acid sequence of TG2 was analyzed using Clustal W (http://www.ebi.ac.kr/Tools/clustalw2/index.html).

#### Protein data bank accession code

The coordinates and structure factors have been deposited in the protein data bank (PDB) under accession code 4PYG.

## Results and Discussion

### Overall structure of GTP-bound TG2

**Figure 1:**
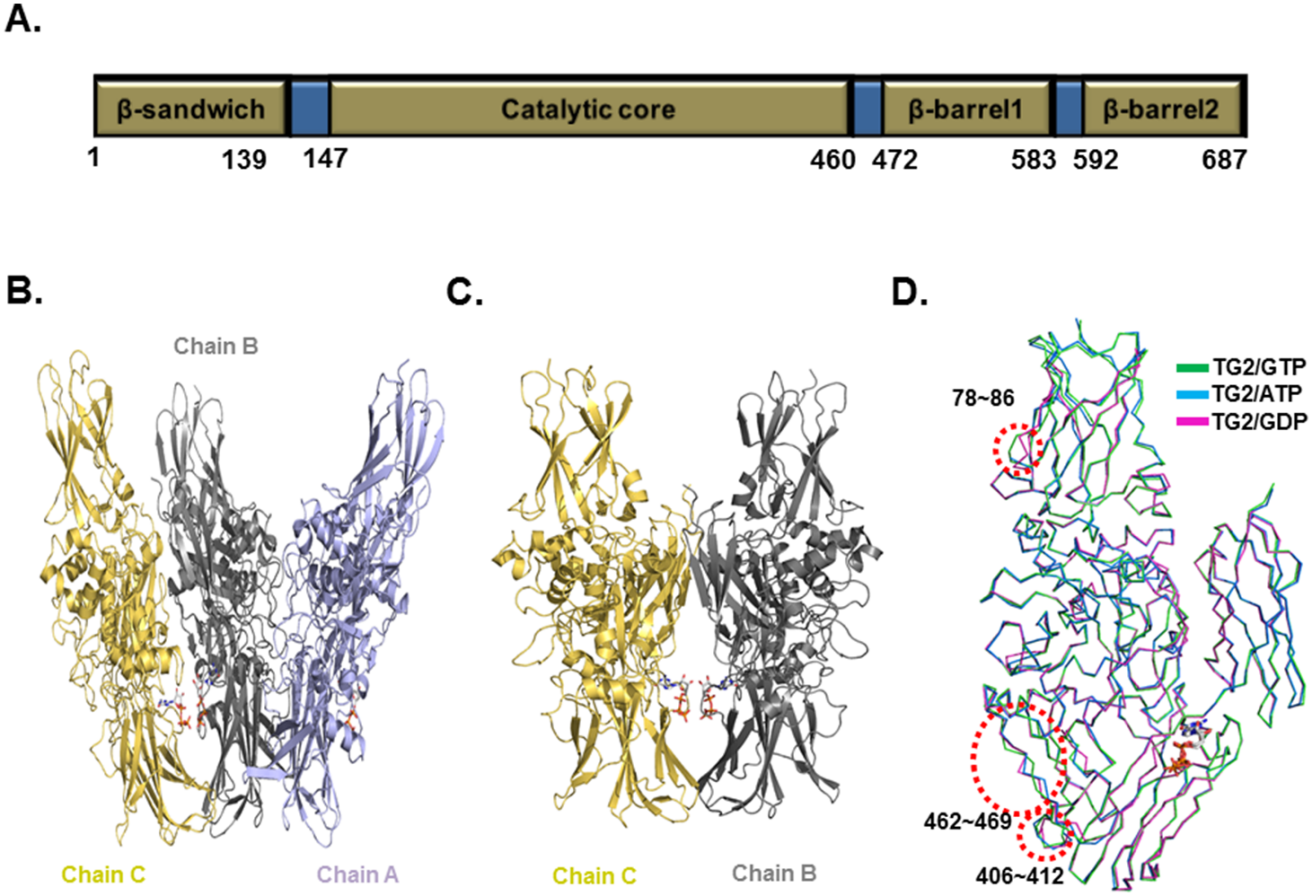
Crystal structure of TG2 in complex with GTP. A. Domain boundary of TG2. Domains are gold and the amino acid residue numbers corresponding to each domain are indicated. B. Ribbon diagram of the structure of TG2 in complex with GTP. Three molecules in the asymmetric unit are shown (Chain A, Chain B and Chain C). C. Symmetric dimer formed by Chain B and Chain C is shown. D. Superposition of the structure of the TG2/GTP complex with known structures. Structurally different regions are indicated by red-dot circles.

Human TG2 consists of four domains, the N-terminal β-sandwich domain, catalytic domain and two C-terminal β-barrel domains (Figure 1A). GTP-bound TG2 was purified. The 2.8 Å crystal structure of the full-length TG2 was solved and refined to an *R*_work_ = 20.6% and *R*_free_ = 28.9%. The data collection and refinement statistics are summarized in Table 1. There were three monomers in the asymmetric unit, chain A, chain B, and chain C (Figure 1B). Chain A was independently located, while chain B and chain C form a symmetric dimer that is similar with the previously reported structure of GDP-bound TG2 (Figure 1C). The final model contained residues 4-688 for the A, B and C chains. The model of the three N-terminal residues was not built because of the poor electron density map. Residue Leu688 is an extra residue from the cloning construct. All three chains are identical and contain GTP (Figure S1). The high resolution structure showed that the overall structure is similarwith the previously reported structure of GDP- or ATP- bound TG2. However, our current structure contained GTP, which is a natural inhibitor of TG2. The root mean square deviations (R.M.S.D.) between GTP-bound TG2 (current form) and ATP-bound TG2 or GDP-bound TG2 are 0.62 Å and 0.65 Å, respectively. A superposition study for structural comparison between TG2 with different nucleotides showed that amino acids 78–86, 406–412, and 462–469 were structurally different, indicationg that those three regions are flexible in solution (Figure 1D).

**Table 1:** Crystallographic statistics.

Data collection	Native
Space group	*C222_1_*
Cell dimensions	
* a*, *b*, *c*	133.54 Å, 216.21 Å, 165.18 Å
Resolution	50–2.8 Å
† *R*_sym_	12.5% (42.8%)
†Mean I/σ(I)	22.4 (4.2)
†Completeness	99.9% (100%)
†Redundancy	6.5 (6.5)
**Refinement**	
Resolution	50−2.8 Å
No. reflections used	29,223
*R*_work_/*R*_free_	20.6%/28.9%
No. atoms	
Protein	16,257
Water and other small molecules	205
Average B-factors	20.6 Å^2^
R.M.S deviations	
Bond lengths	0.019 Å
Bond angles	1.966°
Ramachandran Plot	
Most favored regions	90.2%
Additional allowed regions	9.8%

† Highest resolution shell is shown in parenthesis.

### Stoichiometry of TG2 in solution

**Figure 2:**
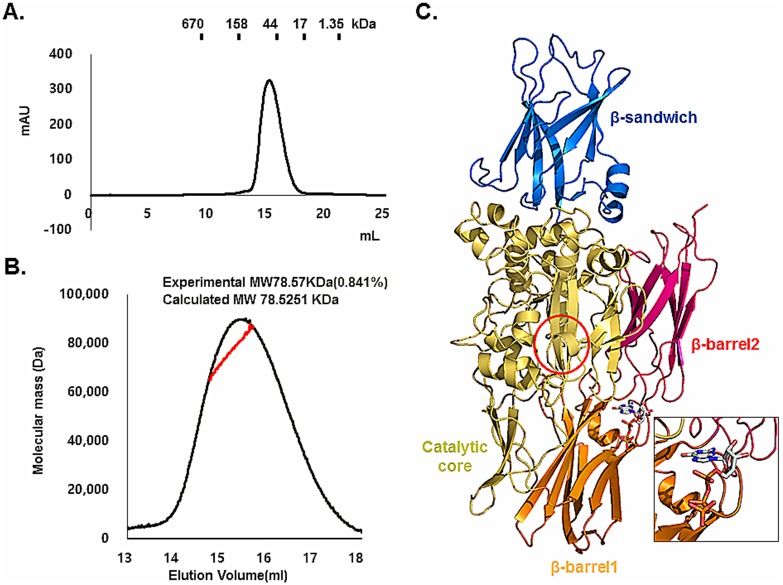
Stoichiometry of TG2 in solution. A. Gel-filtration chromatogram of TG2. A profile obtained from TG2 in buffer containing 20 mM Tris-HCl at pH 8.0 and 150 mM NaCl is shown. Protein size markers are indicated. B. Multi-angle light scattering (MALS) measurement of TG2 in complex with GTP. The x-axis and y-axis indicate the elution volume and molecular mass, respectively. C. Monomeric structure of TG2 in complex with GTP. The active site is shown by a red circle. The GTP binding site is magnified for better visualization.

Previous biochemical and structural studies showed that TG2 could exist as a monomer or dimer in solution. ATP-bound TG2 reportedly exists as monomer, while the structure of GDP-bound TG2 was solved as a dimer [29], [30]. Based on this, the stoichiometry of TG2 in solution is controversial. Upon gel-filtration chromatography, GTP-bound TG2 was eluted at around 14 ml, which corresponds to ∼80 kDa, indicating that it exists as a monomer (Figure 2A). To analyze the stoichiometry of GTP-bound TG2 in solution more accurately, we conducted multi-angle light scattering (MALS). The theoretically calculated molecular weight of the monomeric TG2, including the C-terminal His-tag and GTP, was 78.52 kDa, and the experimental molecular weight from MALS was 78.57 kDa (0.84% fitting error), with a polydispersity of 1.0 (Figure 2B). Based on our analysis using gel-filtration chromatography and MALS, we concluded that GTP-bound TG2 exists as a monomer in solution. Our current monomeric structure of TG2 showed that all four domains are well oriented and that GTP was located in the GTP binding pockets created by two β-barrel domains and the catalytic domain (Figure 2C). The catalytic triad is masked by two β-barrel domains, indicating that this structure is a typical closed form of TG2.

### Structural analysis of GTP specificity for TG2

**Figure 3:**
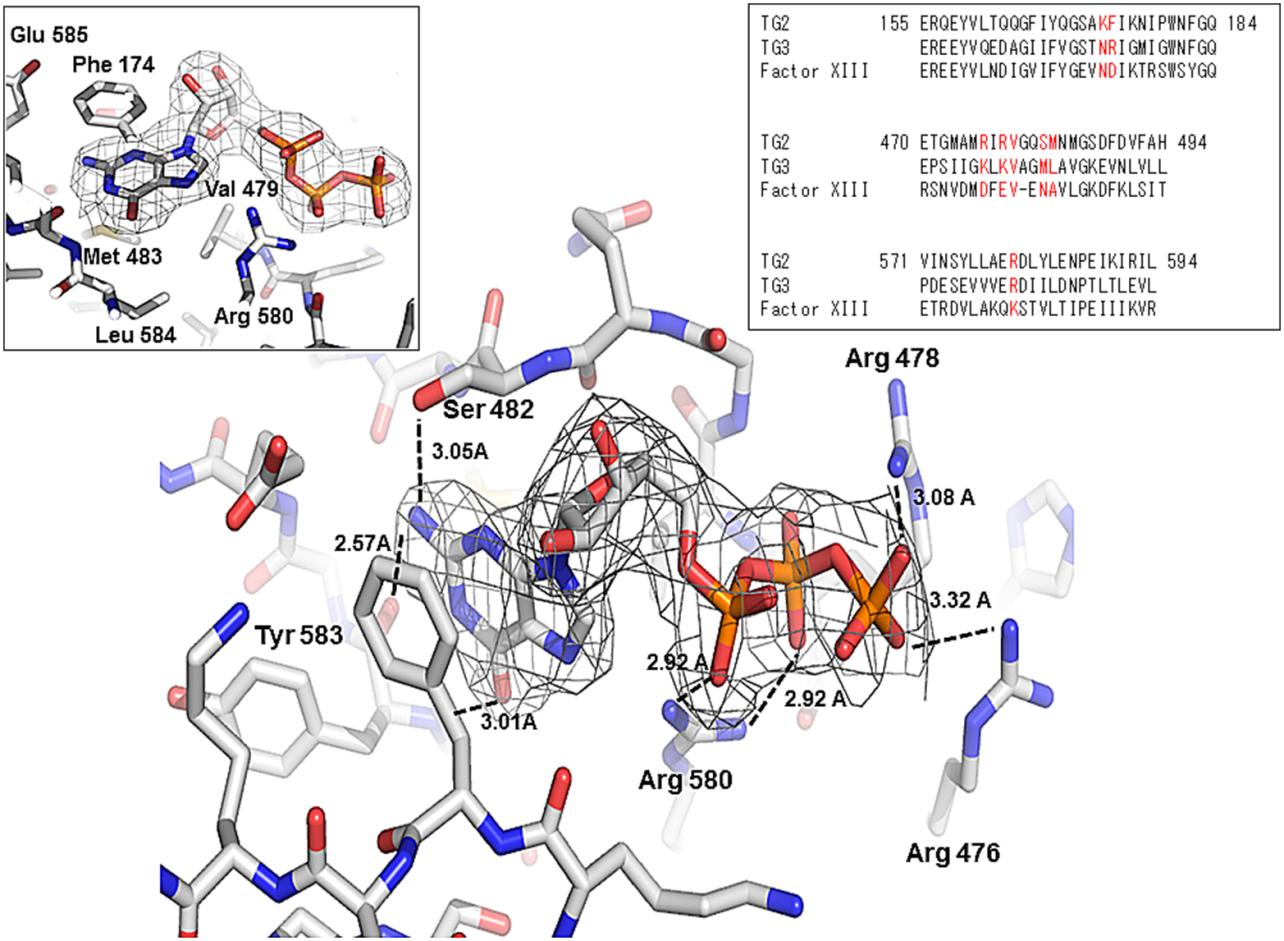
Environment of the GTP binding site in TG2. An omit density map contoured at the 1-σ level around GTP. The residues involved in the GTP interaction are indicated. H-bonds are shown as black-dashed lines. The whole GTP binding site is shown in the left panel. Sequence alignment between TG2, TG3, and Factor XIII for sequence comparison of the GTP binding site is shown in the right panel.

**Figure 4:**
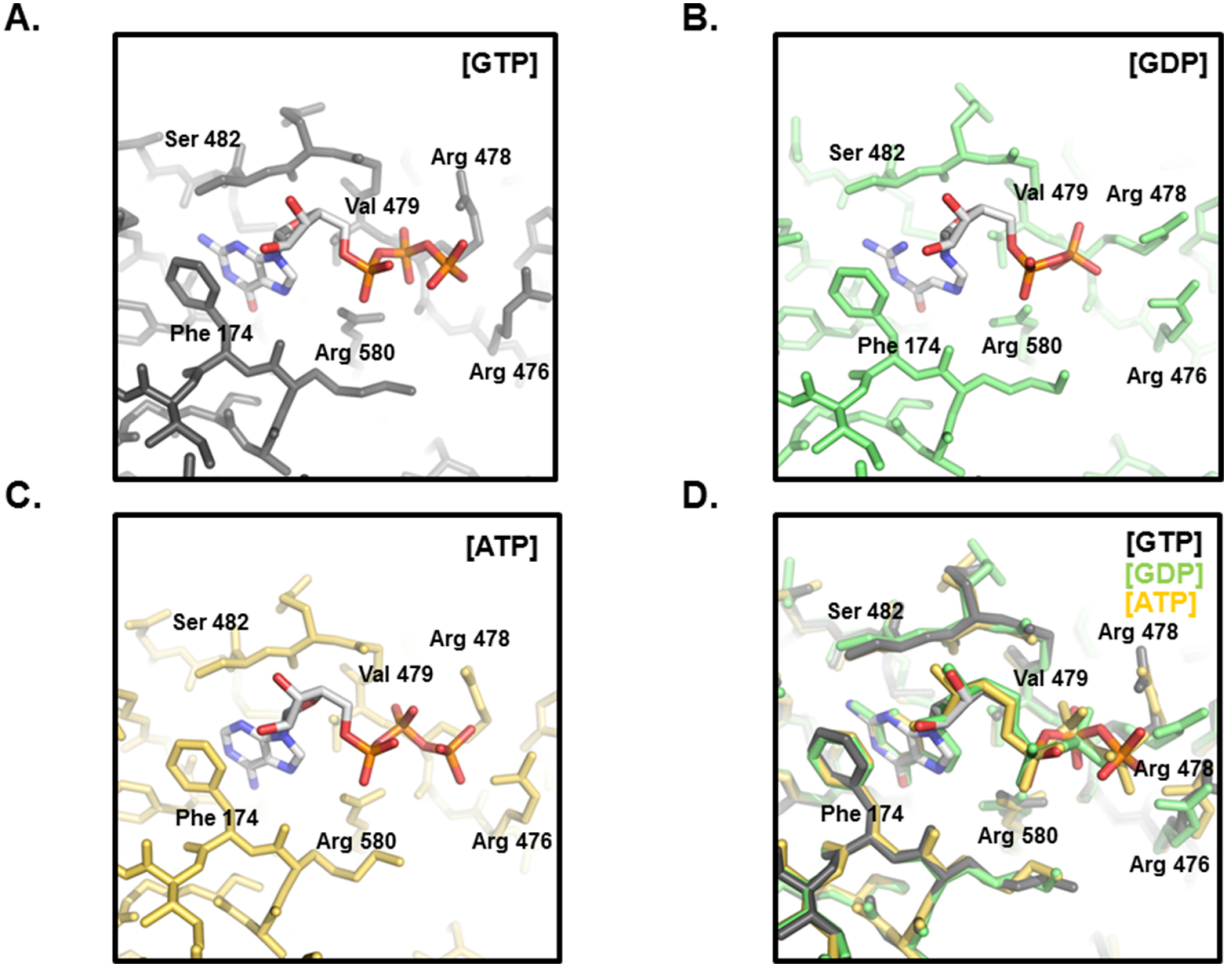
Comparison of GTP binding sites. A. Environment of the GTP binding site of the TG2/GTP complex. Critical residues for the formation of the GTP binding site are indicated. B. Environment of the GTP binding site of the TG2/GDP complex. C. Environment of the GTP binding site of the TG2/ATP complex. D. Superposition of the GTP binding site of the TG2/GTP complex with the TG2/ATP complex and the TG2/GDP complex.

The unique GTP binding site on TG2 is located in a cleft between the catalytic core and the first β-barrel. TG2 interacts with GTP in the absence of Mg^2+^. Although other GTP binding proteins such as many small G proteins contain serine/threonine residues that bind to the phosphates of GTP and participate in Mg^2+^ ion coordination, TG2 does not contain them. Several positively charged amino acids, Arg476, Arg478, and Arg580, surround the negatively charged phosphate moieties of the GTP instead (Figure 3). Arg580 most strongly interacted with GTP by forming two hydrogen bonds (H-bonds) (Figure 3). The importance of Arg580 for the interaction with GTP has been previously revealed in a mutation study [37], [38]. Specifically, mutation of Arg579 in rat TG2 (Arg580 in human TG2) abolished GTP binding [37], [38] and renders the transamidase activity of the protein insensitive to inhibition by GTP [37]. Two more positively charged residues, Arg476 and Arg478, also contribute to accommodate GTP by forming H-bonds with γ-phosphate of GTP. Besides charged interactions and H-bonds, hydrophobic interactions formed by Phe174, Met483, and Val479 from TG2 are important for stabilizing the guanine moiety on GTP (Figure 3). All residues that are critical for GTP binding are conserved in TG3, but not in Factor XIII, indicating that GTP affects the activity of TG3, but not Factor XIII. In fact, previous biochemical studies showed that TG3 activity is inhibited by GTP. However, Factor XIII activity in blood coagulation was not inhibited by GTP [39]–[42]. The majority of the residues contacting GTP come from the end of the first β-strand of the first β-barrel domain and the loop that connects it to the next β-strand. Only two residues from the catalytic core, Lys173 and Phe174, contribute to the GTP interaction. H-bonds formed by Ser482 and Tyr583 can be formed with GTP, but not with ATP, indicating that GTP binds more strongly to TG2. This tight interaction of GTP to TG2 explains why it can more efficiently inhibit the activity of TG2 than ATP. Comparison of the TG2/ATP and TG2/GDP complexes revealed that the locations of the purine moiety of nucleotides are almost the same (Figure 4A, B, C, D). Arg478 was moved outside in the TG2/GTP complex and TG2/ATP complex, while Arg478 was located inside to form a stable H-bond with β-phosphate of GDP. The large movement of Arg478 upon accommodation of GTP or ATP occurs due to sterical collision with the γ-phosphate group (Figure 4D). The location of Arg478 was also unique at the TG2/GTP complex. The side-chain of Arg476 on the structure of the TG2/GTP complex was located on the outside from GTP, while it was located inside on the TG2/ATP and TG2/GDP complexes.

### Formation of inter-molecular disulfide bond between Cys230 and Cys370

**Figure 5:**
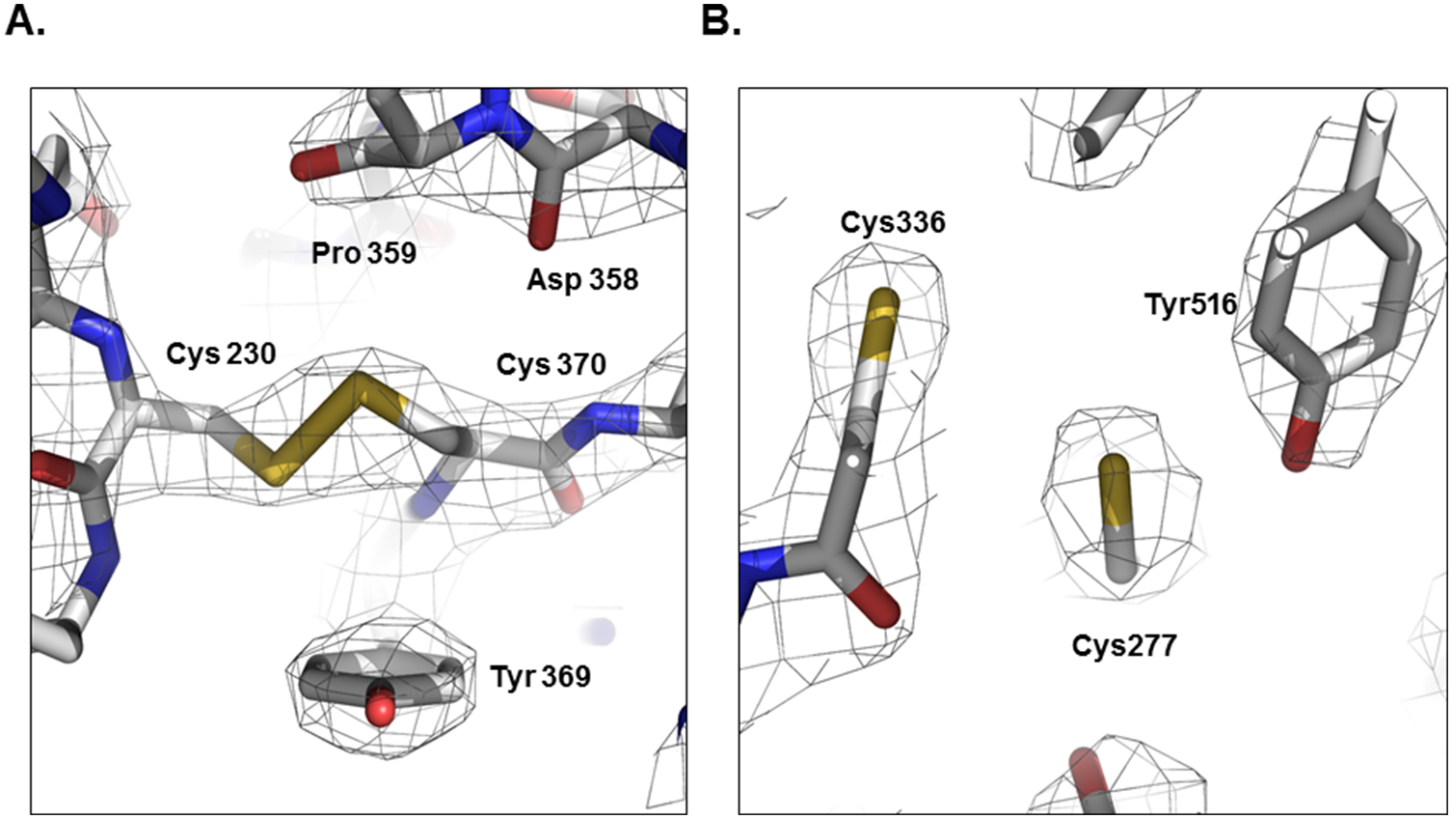
The condition of cysteines at the redox sensor and active site. A. Cys230 and Cys370 at the redox sensor in TG2 formed a disulfide bond, even under highly reduced conditions. B. Cys277 and Cys336 at the active site in TG2 did not form a disulfide bond.

TG2 activity is also regulated by redox potential [43]. Under reducing conditions, the activity of TG2 is increased, while it is inhibited by oxidative conditions. Previous studies identified a redox-sensitive cysteine triad consisting of Cys230, Cys370, and Cys371 [43] that is involved in oxidative inactivation of TG2. It is known that Cys370 can form disulfide bonds with both Cys230 and Cys371 [43]. The disulfide bonds formed by cysteine triads under oxidative conditions inactivate TG2. Cys230, which is only conserved among TG2 family members and considered a TG2 specific redox sensor, facilitates formation of a Cys370–Cys371 disulfide bond via an intermediate Cys230–Cys370 disulfide bond. A Cys370–Cys371 disulfide bond was only observed in the open active form of TG2 [31], while the Cys230–Cys370 disulfide bond was only detected in the TG2/ATP complex, and not the TG2/GDP complex [29], [30]. Cys370–Cys371 disulfide bond was not detected at either the TG2/ATP or TG2/GDP complex. We observed a Cys230–Cys370 disulfide bond in our current TG2/GTP complex structure (Figure 5A). Interestingly, this disulfide bond was formed under highly reduced conditions because the final crystallization condition contains 5 mM DTT in solution. This may indicate that Cys230–Cys370 disulfide bond is formed in the presence of GTP, even under highly reduced conditions, and TG2 becomes active in the absence of GTP and under reduced conditions, when it is possible for the Cys370–Cys371 disulfide bond to be formed. Although there is a cysteine residue (Cys336) near Cys277, which is an active site cysteine, Cys277 was reduced (Figure 5B). It may be possible to form a disulfide bond between Cys336 and Cys277 under oxidative conditions, preventing the activity of TG2.

### Evidence of GTP binding site evolution in TG2

**Figure 6:**
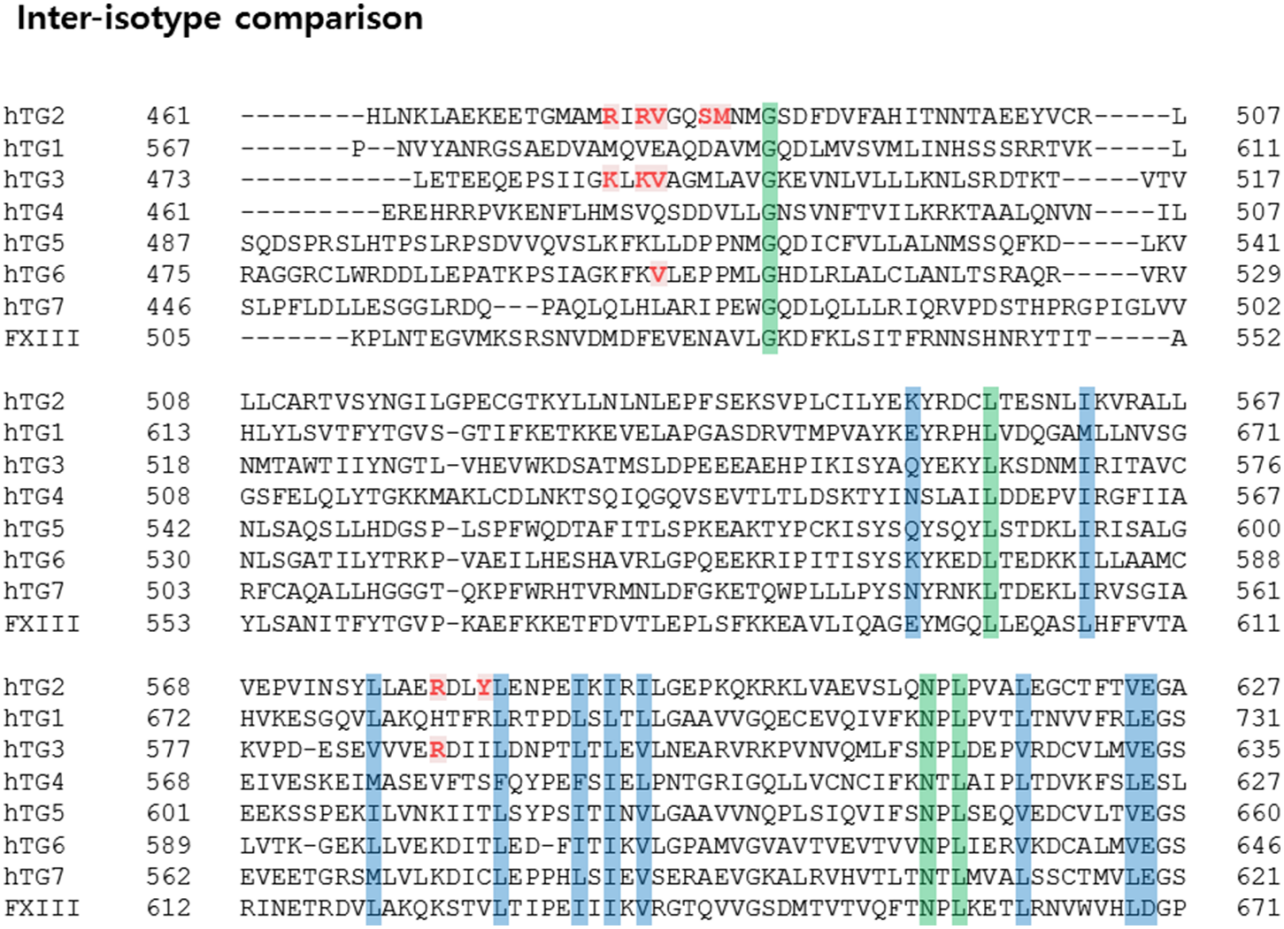
Sequence alignment between inter-isotype TG family members. Amino acid residues conserved at the GTP binding site are shown in red. Amino acid residues perfectly and partially conserved across the isotype are highlighted with green or blue, respectively.

**Figure 7:**
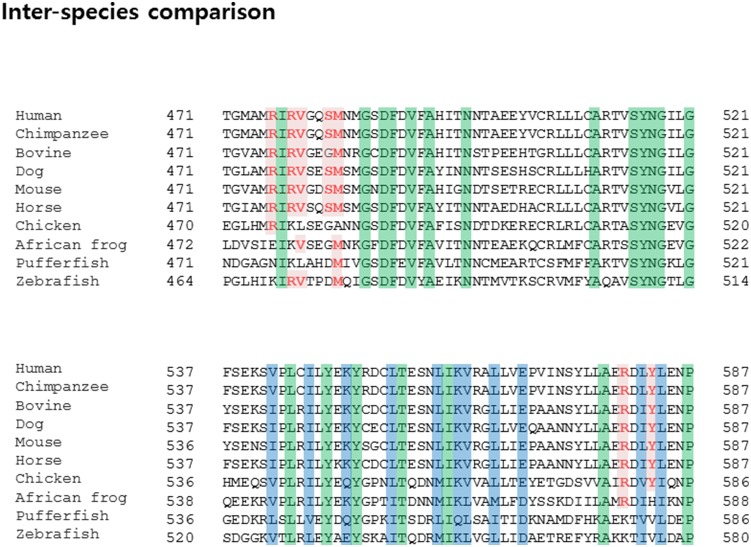
Sequence alignment between inter-species TG2 family members. Conserved GTP-binding amino acid residues are highlighted in red. Amino acid residues perfectly or partially conserved across the species are highlighted ingreen orblue, respectively.

Although the TG family shares similar catalytic mechanisms, the activity of individual members is regulated differently. For example, TG2, TG3 and TG4, but not TG1 and Factor XIII, are inhibited by GTP [39]–[42], [44], [45]. To investigate the reason for this, we compared the sequence of TG family members and analyzed amino acid residues critical for binding to GTP. As expected, Arg476, Arg478, Val479, Ser482, Met483, Arg580, and Tyr583 on TG2 are not conserved on the TG1 and Factor XIII (Figure 6). In the case of Factor XIII, Lys173 and Phe174 are replaced by Asn and Asp, which are not related at all. In addition, Arg476 and Arg478 are replaced by Asp and Glu, which are opposite charged amino acids. This alignment clearly indicates that GTP cannot bind to Factor XIII, and the activity of Factor XIII cannot be regulated by GTP. TG3 contains several conserved residues critical to GTP binding that TG4 does not contain. Accordingly, further studies are needed to determine how TG4 activity can be regulated by GTP without a distinct GTP binding site. Inter-isotype sequence comparison indicates that several families, including TG2 and TG3, contain conserved GTP binding sites, while others do not. GTP can influence the activity of a TG family that contains a GTP binding pocket. Interestingly, sequence comparison between inter-species showed that GTP binding pockets formed by the Arg476, Arg478, Val479, Ser482, Met483, Arg580, and Tyr583 residues are well conserved in mammals, but not in birds, frogs, or fish (Figure 7). This pattern of conservation may reflect the evolutionary scenario of GTP binding to TG2. Initially, GTP was not involved in controlling the activity of TG2. However, as evolution advanced, TG2 began to use GTP. The benefit of GTP usage is not yet known; therefore, future studies investigating the evolutionary aspects of TG2 are warranted.

## Supporting Information

Figure S1 Superposition of three chains in the asymmetric units.
(TIF)
