# Supplementary figures and images for: Crystal Structure of Transglutaminase 2 with GTP Complex and Amino Acid Sequence Evidence of Evolution of GTP Binding Site
Source: PLoS One. 2014 Sep 5;9(9):e107005. doi: 10.1371/journal.pone.0107005 (PMC4156391; doi:10.1371/journal.pone.0107005)

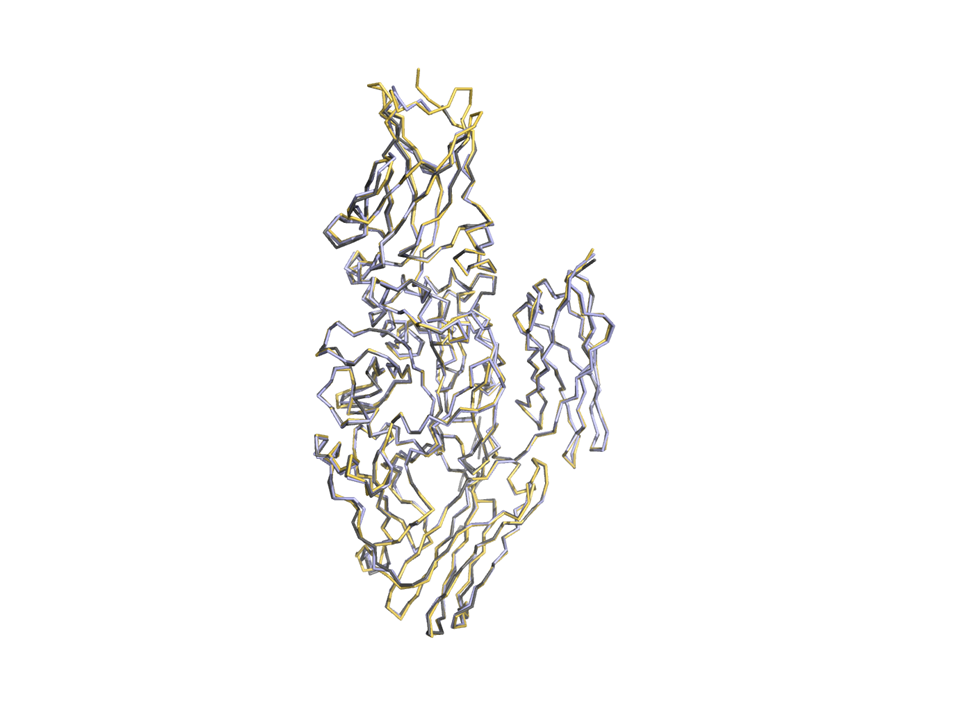

Supplement: Figure S1 — Superposition of three chains in the asymmetric units. (TIF) [file pone.0107005.s001.tif]
